# Supplementary material for: Complexome profiling of the Chlamydomonas psb28 mutant reveals TEF5 as an early PSII assembly factor
Source: Plant Cell. 2025 Mar 17;37(6):koaf055. doi: 10.1093/plcell/koaf055 (PMC12164590; doi:10.1093/plcell/koaf055)
Supplement: koaf055_Supplementary_Data [file koaf055_supplementary_data.zip › psb28_interactive.zip!/plots/intensity/qualityPlots/peptides/PSBR_Cre06.g261000.t1.2.html]

### Cre06.g261000.t1.2

Trivial name: PSBR  
SeqCoverage [median]: 53.90  
Mass: 15.14 kDa  
  
 

| Sequence | MissedCleavage | Mass [Da] | wtIntensity | muIntensity | Score |
| --- | --- | --- | --- | --- | --- |
| DWKDASGR | 1 | 933.43 | 1414.97 | 0.00 | 91.85 |
| DWKDASGRK | 2 | 1061.53 | 5201.89 | 0.00 | 96.34 |
| FMNKKDWK | 2 | 1095.55 | 13459.93 | 0.00 | 68.85 |
| FMNKKDWKDASGR | 3 | 1581.77 | 906.38 | 0.00 | 72.50 |
| GYGVYR | 0 | 713.35 | 19215.44 | 631.77 | 130.51 |
| GYGVYRYEDK | 1 | 1248.58 | 602.31 | 0.00 | 64.30 |
| KDWKDASGR | 2 | 1061.53 | 16339.20 | 1004.90 | 131.45 |
| QNLMGK | 0 | 689.35 | 4671.72 | 0.00 | 120.06 |
| TDITKVGLNSIEDPVVK | 1 | 1827.00 | 23285.94 | 2033.61 | 156.11 |
| TDITKVGLNSIEDPVVKQNLMGK | 2 | 2498.34 | 18302.26 | 0.00 | 105.06 |
| VGLNSIEDPVVK | 0 | 1268.70 | 2593657.84 | 625247.78 | 333.88 |
| VGLNSIEDPVVKQNLMGK | 1 | 1940.04 | 426775.55 | 213504.29 | 236.75 |
| YEDKYGANVDGYSPIYTPDLWTESGDSYTLGTK | 1 | 3704.67 | 2137.80 | 0.00 | 62.92 |
| YGANVDGYSPIYTPDLWTESGDSYTLGTK | 0 | 3169.44 | 3991.90 | 0.00 | 103.21 |
|  |  |  | 3129963.1 | 842422.4 |  |
